# A Clinical Viewpoint on the Use of Targeted Therapy in Advanced Gastric Cancer

**DOI:** 10.3390/cancers15225490

**Published:** 2023-11-20

**Authors:** Magdalena Skórzewska, Katarzyna Gęca, Wojciech P. Polkowski

**Affiliations:** Department of Surgical Oncology, Medical University of Lublin, Radziwiłłowska 13 St., 20-080 Lublin, Poland; wojciech.polkowski@umlub.pl

**Keywords:** targeted therapy, immunotherapy, gastric cancer

## Abstract

**Simple Summary:**

Outcomes for patients with advanced gastric cancer continue to be unsatisfactory despite the inclusion of new targeted therapies in the treatment options. The effectiveness of targeted therapies is limited for a particular subset of the population. This is related to the fact that specific biomarkers are present in a very small percentage of patients with advanced gastric cancer. It is paramount to conduct further research focused on identifying new molecular targets, as this can significantly improve the effectiveness of advanced-stage therapy.

**Abstract:**

The development of therapies for advanced gastric cancer (GC) has made significant progress over the past few years. The identification of new molecules and molecular targets is expanding our understanding of the disease’s intricate nature. The end of the classical oncology era, which relied on well-studied chemotherapeutic agents, is giving rise to novel and unexplored challenges, which will cause a significant transformation of the current oncological knowledge in the next few years. The integration of established clinically effective regimens in additional studies will be crucial in managing these innovative aspects of GC. This study aims to present an in-depth and comprehensive review of the clinical advancements in targeted therapy and immunotherapy for advanced GC.

## 1. Introduction

Over the past few decades, a noticeable decrease has been observed in the incidence of gastric cancer (GC) [1,2,3]. Despite the advancements achieved, GC continues to be a major public health issue, especially in East Asia [4,5]. GC accounted for over one million cases and caused 768,000 fatalities globally in 2020. This made it the third leading cause of cancer-related deaths and the fifth most commonly diagnosed cancer worldwide [6].

For patients with advanced and metastatic GC, targeted therapy is a promising treatment option. The approach comprises the identification of molecular targets in cancer cells and the development of drugs capable of blocking or inhibiting these targets [7]. The effectiveness of targeted therapy surpasses traditional chemotherapy (CTH) as it meticulously targets cancer cells while leaving normal cells unharmed, leading to a reduction in side effects [8]. Several targeted therapy drugs have been approved for the treatment of advanced or metastatic GC, and ongoing research continues to identify new targets and develop new drugs [9]. The objective of personalized medicine is to offer each patient the most suitable treatment based on their specific circumstances. Thus, the determination of the molecular characteristics of each patient’s tumor can assist in the selection of targeted therapy drugs that are more likely to be effective [10].

GC’s prognosis is unfavorable due to its frequent diagnosis at an advanced stage. The establishment of targeted treatment options necessitates the identification of specific biomarkers that concurrently serve as therapeutic targets [9]. Although new targeted therapies have been added to the treatment portfolio, outcomes for patients with advanced GC remain unsatisfactory. The effectiveness of targeted therapies is limited for a particular subset of the population. This is related to the fact that specific biomarkers are present in a very small percentage of patients with advanced GC [10]. It is of utmost significance to conduct additional research aimed at discovering novel molecular targets, as this can greatly enhance the efficacy of therapy in advanced stages.

The objective of this study is to present a comprehensive and detailed review of the clinical developments in targeted therapy and immunotherapy for advanced GC.

## 2. Molecular Classification of Gastric Cancer

**Figure 1:**
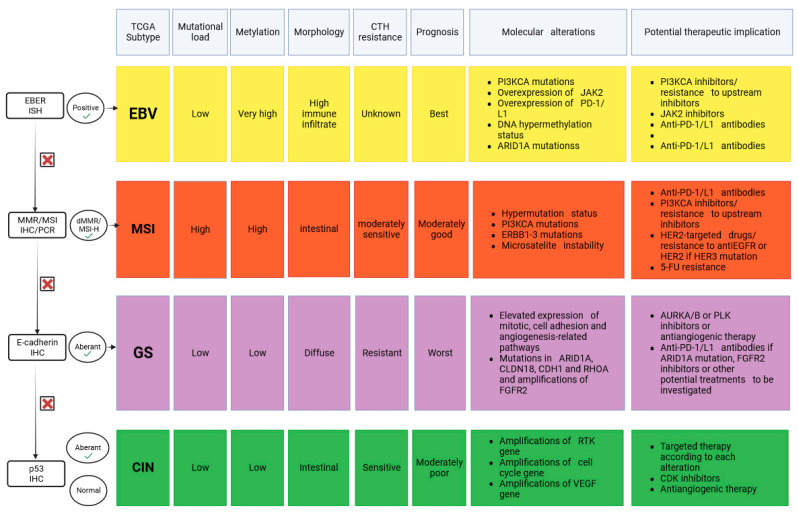
Molecular classification of gastric cancer. CTH—chemotherapy, EBER—EBV-encoded RNA, ISH—in situ hybridization, (d) MMR—(deficient) mismatch repair, MSI-(H)—microsatellite instability (high), IHC—immunohistochemistry, PCR—polymerase chain reaction, p53—tumor protein P53, PIK3CA—phosphatidylinositol-4,5-bisphosphate 3-kinase catalytic subunit alpha, JAK2—Janus kinase 2, HER—human epidermal growth factor receptor, ARID1A—AT-rich interactive domain 1A, PD-1/L1—programmed death-1/ligand1, ERBB1-3—v-erb-b2 avian erythroblastic leukemia viral oncogene homolog 1-3, 5-FU—5-fluorouracil, CLDN18—claudin-18, CDH1—cadherin-1, RHOA—Ras homolog family member A, FGFR2—fibroblast growth factor receptor 2, RTK—receptor tyrosine kinase, VEGF—vascular endothelial growth factor, CDK—cyclin-dependent kinases.

In 2014, The Cancer Genome Atlas (TCGA) Research Network presented the most comprehensive molecular characterization of GC [11]. The study was successful in providing a roadmap that can be used for patient stratification and as a guide for targeted therapy trials. As part of the TCGA project framework, an evaluation was undertaken on 295 primary GC using six molecular platforms, comprising array-based somatic copy number analysis, whole-exome sequencing, array-based DNA methylation profiling, messenger RNA sequencing, microRNA sequencing, and reverse-phase protein array (RPPA). The microsatellite instability (MSI) testing was performed on all tumors. A molecular classification strategy was introduced in this research to divide GC into four subtypes, namely tumors positive for Epstein–Barr virus (EBV) subtype with extreme DNA hyper-methylation (8.8% of the samples), microsatellite-unstable (MSI) subtype with elevated mutation rates and hyper-methylation (21.7% of the samples), genomically stable (GS) tumors with less distinctive genomic alterations (19.7% of the samples), and tumors with chromosomal instability (CIN) with marked aneuploidy and frequent focal amplification of receptor tyrosine kinases (RTKs) (49.8% of the samples) [11]. Figure 1 displays the characteristics of molecular classification.

## 3. Current Targeted Therapies Options for Advanced Gastric Cancer

**Figure 2:**
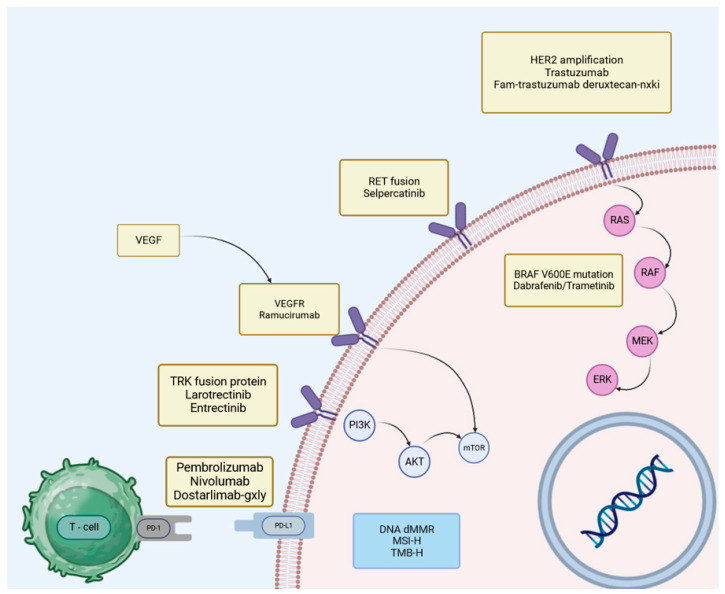
Molecular targets of gastric cancer. PD-1/L1—programmed death-1/ligand1, dMMR—deficient mismatch repair, MSI-H—microsatellite instability (high), TMB-H—tumor mutational burden (high), PIK3CA—phosphatidylinositol-4,5-bisphosphate 3-kinase catalytic subunit alpha, AKT—tropomyosin receptor kinase, mTOR—mammalian target of rapamycin, TRK—tropomyosin receptor kinase, VEGF(R)—vascular endothelial growth factor (receptor), RET—rearranged during transfection, HER2—human epidermal growth factor receptor 2, BRAF—v-raf murine sarcoma viral oncogene homolog B1, RAS—rat sarcoma virus, RAF—rapidly accelerated fibrosarcoma, MEK—mitogen-activated protein kinase, ERK—extracellular signal-regulated kinase.

Targeted therapies have revolutionized the treatment of different solid tumors over the past decade, significantly altering the treatment approach [12]. The swift progress in genomics, proteomics, and transcriptomics has resulted in the recognition of novel molecular modifications and immune phenotype markers that are common among diverse tumor types, regardless of their origin [13]. The approval of drugs known as “histology-agnostic” has been made possible by the availability of pharmacological agents that target such alterations and markers specifically and selectively [14]. A paradigm shift in cancer treatment has been established by this new therapeutic approach, which has paved the way for a new class of biomarker-driven anticancer agents that go beyond tumor histologies [15]. Since 2017, the Food and Drug Administration (FDA) has approved six anticancer agents with a “histology-agnostic” indication: two immune checkpoint inhibitors (ICIs) for cancers with high tumor mutational burden (TMB) or mismatch repair deficiency/microsatellite instability (dMMR/MSI) [16,17,18] and four targeted therapies for tumors harboring a BRAF V600E mutation or a neurotrophic tyrosine receptor kinase (NTRK) gene fusion [19,20,21]. Figure 2 presents the mechanism of action of current therapy options.

## 4. HER2 Overexpression in Gastric Cancer

The overexpression of human epidermal growth factor receptor 2 (HER2) is a molecular anomaly that has been increasingly recognized as a crucial factor in the development and treatment of GC. Involved in cell growth and division, the protein HER2 is a typical component of cell surfaces. In cases of HER2 overexpression, the amplification of the gene responsible for protein production results in an excessive amount of HER2 protein that contributes to the proliferation and metastasis of cancer cells [22,23].

### 4.1. Prevalence and Pathogenesis of HER2 Overexpression in Gastric Cancer

First discovered in 1984, HER2 (also known as Neu and ErbB2) is a proto-oncogene located on chromosome 17. As a member of the HER receptor family, HER2 plays a crucial role in signal transduction pathways that regulate cell growth, differentiation, and progression [22,23,24]. The HER family includes four members, namely HER1 or EGFR, HER2, HER3, and HER4, all of which exhibit an extracellular domain (ECD), a transmembrane domain, and an intracellular kinase domain [22,25]. The absence of ligand-binding activity in HER2 causes its heterodimerization with other family members (HER1 and/or HER3) for activation [26]. Among the various HER signaling dimers, the HER2-HER3 heterodimer stands out as the most active and plays a crucial role in the oncogenic transformation of HER2-driven tumors. The growth and progression of GC tumors are also significantly impacted by the interaction of HER2 overexpression with other signaling pathways. Tyrosine kinase autophosphorylation and heterodimerization by the HER-2 receptor initiate signal transduction, which prompts downstream pathways, among which are the PI3K-AKT and Ras/MAPK pathways [22]. Upon activation, these pathways can lead to uncontrolled cell growth and tumor progression, as they regulate programmed cell death, proliferation, survival, and differentiation [22,23,27]. The evidence indicates that HER2 can hinder cell apoptosis and facilitate proliferation, making a significant contribution to the survival and aggressiveness of cancer cells but also the development of GC [28]. Genetic mutations that result in the overexpression of the HER2 protein and the amplification of the HER2 gene are key mechanisms that contribute to HER2 overexpression in GC [29,30].

The incidence of HER2 overexpression or amplification in GC varies depending on the study, region, population, and testing method. The estimated percentage ranges from 4.4% to 53.4%, with an average of 17.9%. However, among Chinese patients with GC, it is only 8.8% [22,29,31]. HER2 overexpression has been found to be more prevalent in certain subtypes of GC, specifically intestinal type, when compared to diffuse or mixed type, with rates of 31% and 6%, respectively [22,24,32]. The rate of HER2 overexpression is higher in gastroesophageal junction cancer (GEJ) than in gastric corpus cancer (32.2% vs. 21.4%). A potential association could exist between the prevalence of HER2 overexpression and the histologic grade of the tumor, particularly distinguishing between well/moderately differentiated and poorly differentiated [33,34]. HER2 overexpression was also found to be associated with factors such as tumor location, tumor differentiation, Bormann classification, Lauren’s classification, lymph node status, venous invasion, and lymphatic invasion. However, previous studies have shown no correlation with gender, age, or clinical stage [35,36,37,38].

### 4.2. Diagnosis of HER2 Overexpression in Gastric Cancer

Determining the HER2 status of patients with GC is crucial for devising a treatment plan. There are several techniques to evaluate HER2 status, such as immunohistochemistry (IHC), fluorescence in situ hybridization (FISH), and chromogenic in situ hybridization (CISH) [27]. Considering its low cost and ease of implementation, IHC is the most frequently utilized approach for detecting HER2 overexpression in GC. However, it has exhibited a lesser precision than FISH and CISH [22]. Consequently, FISH and CISH are regarded as more reliable techniques for detecting HER2 overexpression in GC [39]. IHC assesses the membranous immunostaining of tumor cells, encompassing the intensity and extent of staining, in addition to the percentage of immunoreactive tumor cells. ISH techniques are employed to identify HER2 gene amplification in cancer cells. In 2008, Hofmann et al. fine-tuned the four-tiered scoring system to evaluate HER2 status in GC, incorporating a threshold of at least 10% immunoreactive tumor cells. HER2-negative status is assigned based on a scoring system that involves a score of IHC0 or IHC1+. IHC0 shows membranous reactivity in less than 10% of cancer cells, while IHC1+ indicates faint membranous reactivity in at least 10% of cancer cells. If a score of 2+ is obtained, which indicates weak-to-moderate membranous reactivity in at least 10% of cancer cells, the outcome is considered equivocal [27,40]. Samples displaying an IHC 2+ value necessitate an additional FISH or CISH test [27,40]. The FISH/ISH findings are presented as a ratio of HER2 gene copies to CEP17 chromosome centromeres identified in a minimum of 20 cancer cells (HER2:CEP17). The FISH/ISH results can be utilized as an alternative reporting method to present the mean HER2 copy number per cell. The identification of tumors with HER2 overexpression is determined as a 3+ IHC score, which reflects strong membranous reactivity in at least 10% of cancer cells, or a 2+ IHC score and FISH/ISH positivity (indicated by a HER2:CEP17 ratio of ≥2 or an average HER2 copy number of ≥6 signals/cell). HER2 IHC outcomes that are positive (3+) or negative (0 or 1+) do not require additional ISH testing [41].

### 4.3. Treatment Options for HER2 Overexpression in Advanced Gastric Cancer

The knowledge of HER2 status is an increasingly important aspect of the clinical management of GC. HER2 overexpression’s importance in GC treatment is due to its susceptibility to targeted therapies. Given the advent of targeted therapies, identifying whether a tumor overexpressed HER2 is a critical factor in guiding treatment decisions and potentially enhancing patient outcomes. The potential for improving outcomes for patients with HER2-positive GC lies in emerging innovative therapies and treatment approaches [42].

Since its approval in 2010, **trastuzumab**, one of the primary molecularly targeted drugs developed, has been recommended for patients with early and advanced HER2-positive gastric or GEJ cancer. This monoclonal antibody has contributed to better prognostic outcomes [23,43]. The effectiveness of trastuzumab in the perioperative treatment of HER2-positive resectable GC, when used alongside chemotherapy or radiotherapy, is still being investigated in multiple trials [42].

Trastuzumab emerged as a safe and effective treatment alternative for patients with advanced gastric or GEJ adenocarcinoma featuring HER2 overexpression, based on the outcomes of the randomized Phase III ToGA trial [24]. Notably, 594 patients diagnosed with HER2-overexpressed gastric or GEJ adenocarcinoma were enrolled in the study. The patients were classified as either locally advanced, recurrent, or metastatic. The participants were randomly assigned to two groups. Trastuzumab was administered to the first group, in combination with cisplatin and fluorouracil or capecitabine. The administration of CTH was the only treatment given to the second group [24]. With the addition of trastuzumab to CTH, the median overall survival (OS) showed a remarkable improvement, from 11 months to 13.8 months. This study determined that the preferred treatment for HER2-positive advanced gastroesophageal adenocarcinoma patients is a combination of trastuzumab, cisplatin, and fluoropyrimidine. According to a post hoc subgroup analysis, patients with IHC 2+ and FISH-positive or IHC 3+ tumors (*n* = 446) who received trastuzumab and CTH experienced a significant improvement in OS [24]. The effectiveness of trastuzumab in combination with capecitabine and oxaliplatin as a first-line treatment for advanced gastric or GEJ adenocarcinoma with HER2 overexpression (*n* = 45) was assessed in the HERXO Phase II trial [44]. According to the study’s findings, the progression-free survival (PFS) and OS rates were 7.1 and 13.8 months, respectively. The average follow-up period was 13.7 months. The rates of complete response (CR), partial response (PR), and stable disease were observed among the subjects, with rates of 8.9%, 37.8%, and 31.1%, respectively. An analysis of 34 patients with metastatic gastric or GEJ adenocarcinoma who were HER2-overexpression-positive revealed that the combination of trastuzumab with a modified FOLFOX-6 regimen (mFOLFOX-6) was better tolerated than the cisplatin plus fluorouracil regimen. This observation was especially evident in patients who had received no prior treatment for tumors with HER2 overexpression [45]. The treatment exhibited an observed response rate (ORR) of 41%, with a median PFS and OS of 9.0 months and 17.3 months, respectively. For GEJ showing HER2 overexpression, the findings indicate that incorporating trastuzumab with capecitabine and oxaliplatin or mFOLFOX is a safe and efficacious therapeutic strategy [45]. Therefore, it is strongly recommended that patients diagnosed with adenocarcinoma and exhibiting HER2 overexpression be treated with trastuzumab in the initial CTH regimen, along with a fluoropyrimidine and a platinum agent (preferably oxaliplatin due to its lower toxicity compared to cisplatin). The use of an FDA-approved biosimilar in place of trastuzumab is acceptable. Trastuzumab, in combination with other chemotherapeutic agents, may be regarded as a first-line treatment option. It is not advisable to incorporate it in second-line therapy [46].

The inhibition of heterodimerization between HER2 and growth factor receptors is the principal mechanism through which HER2-specific antibodies impede HER2 signaling [27]. The mechanism through which trastuzumab acts in cancer cells lacks a general agreement. However, the data reveal that it not only obstructs HER2 dimerization with other HER family members and promotes endocytosis but also elicits cell-mediated immunity and suppresses angiogenesis [29].

**Fam-trastuzumab deruxtecan-nxki**, an antibody–drug conjugate, is produced by conjugating trastuzumab and a cytotoxic topoisomerase I inhibitor using a cleavable tetrapeptide-based linker. The efficacy and safety of fam-trastuzumab deruxtecan-nxki were assessed in the Phase II DESTINY-Gastric01 trial. This study enrolled 188 patients with advanced or metastatic gastric or EGJ adenocarcinoma, with disease progression after two prior lines of therapy (including trastuzumab) [47].

Participants were allocated to one of two treatment groups at a 2:1 ratio. The first group was administered fam-trastuzumab deruxtecan-nxki, while the second group was given either paclitaxel or irinotecan, as decided by the physician. The ORR among patients who were administered fam-trastuzumab deruxtecan-nxki amounted to 51%, compared to 14% in the CTH group. OS (12.5 vs. 8.4 months; *p* = 0.0097), median PFS (5.6 vs. 3.5 months), and the duration of response (11.3 vs. 3.9 months) were also higher in the fam-trastuzumab deruxtecan-nxki group. However, the treatment was found to be more toxic than chemotherapy. In addition to serious adverse events (SAEs) (Grade 3 or higher), which were primarily hematological toxicities, twelve patients developed interstitial lung disease and pneumonitis related to Fam-trastuzumab deruxtecan-nxki, resulting in one death due to pneumonia.

In line with the FDA approval, fam-trastuzumab deruxtecan-nxki can be deemed as a second-line or subsequent option for treating HER2 overexpression-positive adenocarcinoma patients who have not responded to prior trastuzumab-based therapy. However, careful consideration should be given to patient selection, and patients need to be closely monitored to avoid overtoxicity [47].

### 4.4. Clinical Implications, Prognosis, and Survival Rates for HER2 Overexpression in Advanced Gastric Cancer

Numerous studies have shown that HER2 overexpression is significantly correlated with poor outcomes and has a considerable impact on the prognosis of patients with advanced GC [22,23]. Research suggests that HER2 overexpression is a negative prognostic factor [24,27]. The study conducted by Chua et al. established that HER2 overexpression was associated with lower survival rates and intestinal-type GC. [48]. In a study conducted by Gravalos et al., tumors with HER2 amplification were found to be associated with poor mean survival rates and 5-year survival rates [22]. In addition, a retrospective study involving 108 cases revealed that HER2 overexpression was linked to a less favorable 10-year survival [22]. A study conducted by Kurokawa et al. has shown that HER2 overexpression is an independent prognostic factor for patients with resectable GC regardless of the disease stage. Nonetheless, the prognostic value of HER2 for GC is still a matter of controversy since the survival rates of GC patients with HER2 overexpression are subject to the influence of various factors, and the mechanisms of resistance to HER2-targeted therapy are still being studied [27,42,49,50]. In the course of HER2-targeted therapy, cells with HER2 amplification or overexpression are selectively eliminated, while drug-resistant cells continue to multiply [42]. The resistance to HER2-targeted therapy can be caused by the interactions and cross-signaling between HER2 and other growth factor receptors [51]. Hence, HER2 overexpression not only contributes to tumor growth and progression but also impacts treatment outcomes. The observed interactions between HER2 and other signaling pathways reveal the intricate nature of HER2 overexpression in GC and its impact on both tumor growth and progression.

GC with HER2 overexpression demonstrates more aggressive biological behavior and a higher incidence of recurrences [24]. The specific mechanism underlying this increased aggressiveness is not fully comprehended, but it is hypothesized to involve signaling pathways that facilitate cellular growth and impede programmed cell death [24]. The early identification and treatment of HER2 overexpression are essential for improving outcomes in individuals with GC and can considerably influence survival rates. HER2-targeted therapy has been shown to enhance both the survival and quality of life of patients diagnosed with HER2-positive gastric and GEJ cancers [52].

In conclusion, although HER2 overexpression is most prominently associated with breast cancer, its significance in GC is being increasingly recognized in clinical oncology. Similar to any cancer diagnosis and treatment, individual patient care must be tailored based on a comprehensive assessment, including HER2 status and other molecular markers. It is therefore essential to comprehend the mechanisms of HER2 overexpression and its interactions with other signaling pathways to develop innovative treatment approaches and therapies that can improve the outcomes of patients with HER2-positive GC.

## 5. Immune Checkpoint Inhibitors for Gastric Cancer

### 5.1. Overview of the Mechanism of PD-1/PD-L1 Pathway

The regulation of immune suppression caused by tumors is achieved through the use of advanced molecules, including programmed death-1 (PD-1). PD-1 expression is found in certain immune cells, including T cells, B lymphocytes, and natural killer (NK) cells. The binding of PD-1 to either of its ligands, PD-L1 or PD-L2, induces the suppression of cytokine production from immune cells, comprising interferon-γ (IFN-γ), tumor necrosis factor-α (TNF-α), and interleukin (IL)-2, along with the inhibition of T-cell activation [53]. The significance of this mechanism lies in its ability to prevent autoimmune reactions, which are essential in maintaining physiological conditions. However, cancer cells also use it to elude detection by the immune system [54]. Within the tumor immune microenvironment (TIME), PD-1 is also expressed in tumor-infiltrating lymphocytes (TILs) and plays a role in regulating antitumor immune response [55]. The functional inactivation of TILs is caused by the binding of PD-L1 expressed on the surface of tumor cells to PD-1, which results in the loss of their ability to eliminate tumor cells [56]. Consequently, the PD-1/PD-L1 pathway has been recognized as a negative modulator of immune response by restricting the activity of TILs in the TIME. The functionality of cytotoxic T lymphocytes (CTLs) may be reactivated, and their ability to combat tumor cells may be strengthened through the administration of monoclonal antibodies (mAbs) that impede the PD-1/PD-L1 pathway [57]. From the perspective of current scientific evidence, the immune checkpoint PD-1/PD-L1 pathway is an essential element that contributes to the process of immunoediting, tumor progression, and metastasis [58].

### 5.2. Diagnostic Methods for Immune Checkpoint Inhibitor Therapy

#### 5.2.1. PD-1 and PD-L1 Testing

PD-L1 expression is an established biomarker for determining treatment efficacy in GC [59]. PD and PD-L1 testing can provide valuable information for predicting patient outcomes and tailoring treatment plans for individuals with GC. A study by Lian et al. revealed that PD-L1 expression was associated with poor prognosis in gastric cancer patients [60]. A meta-analysis by Voutsadakis et al. revealed that PD-L1 expression was associated with a better response to immunotherapy [61].

Conducting PD-L1 testing is recommended in order to determine PD-1 inhibitor treatment eligibility for patients with locally advanced, recurrent, or metastatic GC. The process of testing entails the utilization of an FDA-approved companion diagnostic test, which is a qualitative IHC assay that employs anti-PD-L1 antibodies to identify PD-L1 protein levels in formalin-fixed paraffin-embedded (FFPE) tumor tissue. To guarantee the accurate evaluation of the specimen, it is essential that a minimum of 100 tumor cells be present on the slide stained for PD-L1. The PD-L1 combined positive score (CPS) is utilized for the assessment of both the effectiveness of PD-1/PD-L1 inhibitors and as a prognostic factor [62,63]. The calculation of CPS requires dividing the number of cells stained with PD-L1 by the total number of viable tumor cells assessed and then multiplying the quotient by 100 [64]. In the staining process with the PD-L1 antibody, both tumor cells and stromal cells are included [65]. A CPS value that is greater than or equal to 1 signifies PD-L1 expression in a specimen. The threshold value for PD-L1 expression in GC immunotherapy remains the subject of investigation [64]. Tumor proportion score (TPS) is a metric that is presented in certain trials, reflecting the percentage of viable tumor cells exhibiting partial or complete membrane staining at any intensity. The determination of PD-L1 expression in the specimen hinges on the TPS measure, which requires a minimum value of 1% to establish PD-L1 expression and a minimum value of 50% to establish high PD-L1 expression [62,66]. PD-L1 status can be determined on both therapy-naïve biopsies and surgical specimens after neoadjuvant treatment [62,67].

#### 5.2.2. MSI and MMR Testing

In order to determine eligibility for immunotherapy in gastrointestinal cancers, PD-L1 expression is used in combination with MSI/MMR [67]. The molecular characterization of GC necessitates the inclusion of MSI and MMR testing, which delivers vital details for prognosis, treatment selection, and genetic counseling. Microsatellites, which are sequences of DNA, have a length that ranges from one to six repetitions of nucleotides [68]. Both coding and non-coding regions of the genome contain these DNA motifs, which exhibit high levels of polymorphism among populations but remain stable within individuals [68]. The MMR system comprises various proteins, specifically the products of hMLH1, hMSH2, hMSH6, and hPMS2 genes, which supervise the accuracy of DNA replication. The MMR system’s focus is on the identification and correction of replication errors, such as base mismatch, insertions, and deletions, when detected [69,70].

It is recommended that all GC patients diagnosed recently undergo universal testing for MSI via polymerase chain reaction/next-generation sequencing (PCR/NGS) or MMR via IHC. The MSI status is determined through the examination of gene expression levels of microsatellite markers (BAT25, BAT26, MONO27, NR21, and NR24) using PCR. To evaluate MMR deficiency, IHC is employed to examine the nuclear expression of proteins accountable for DNA mismatch repair, namely MLH1, MSH2, MSH6, and PMS2 [71]. PCR/NGS for MSI and IHC for MMR proteins measure separate biological effects resulting from impaired MMR function. The presence of a deficiency in one or more MMR proteins, resulting in a deficient MMR status (dMMR), leads to frameshift mutations that are especially concentrated in microsatellite repeats. Consequently, MSI is regarded as the epiphenomenon of dMMR [72]. In accordance with the guidelines of the College of American Pathologists DNA Mismatch Repair Biomarker Reporting, FFPE tissue is subjected to testing, and the results are interpreted as MSI—high (MSI-H) or dMMR [73]. The susceptibility of patients with dMMR/MSI-H tumors, regardless of their tissue of origin, to ICI stems from differences in the components of TIME. Evidently, several factors within the TIME present differences between dMMR/MSI-H and pMMR/MSI-L tumors, comprising immune cell phenotypes, cytokine networks, and immune checkpoints [74,75,76]. Tumors with MSI-H typically exhibit a higher TMB, which leads to the development of more neo-antigens that can be recognized by the immune system [77]. The TMB, which is measured based on the number of mutations per megabase (muts/Mb) present in cancerous cells, can be determined by employing NGS. Consequently, the TMB proves to be a significant biomarker for patients with dMMR/MSI-H GC in response to anti-PD-1 therapy [78].

Considering the distinctive attributes and clinical behavior of microsatellite instability GC compared to its microsatellite-stable equivalent, it may be appropriate to include an MSI test in the diagnostic process for all tumor stages to guarantee optimal and personalized treatment for each patient [79].

### 5.3. Treatment Options for PD-1- and PD-L1-Positive Advanced Gastric Cancer

In comparison to chemotherapy, PD-1/PD-L1 inhibitors are shown to improve OS in gastric cancer but not PFS [62]. Therapeutic agents that target PD-1 or PD-L1 immune checkpoints have emerged as a promising treatment option for multiple types of cancers, including GC.

**Pembrolizumab**, a PD-1-type antibody, was granted FDA approval in 2017 for the treatment of individuals with solid tumors that are unresectable or metastatic, have the MSI-H or dMMR gene, have undergone progression after previous treatments, and have no other satisfactory alternatives for treatment [18]. This approval represents a major milestone, as it is the first of its kind, unrestricted by tissue or site, and supported by clinical trial data from 149 patients with MSI-H/dMMR cancers who participated in five different trials conducted across multiple centers [80,81]. Pembrolizumab resulted in a response duration of ≥6 months in 78% of those who responded, with an ORR of 39.6% (irrespective of cancer histology). There were 11 CRs and 48 PRs.

Pembrolizumab was approved by the FDA in June 2020 for treating metastatic TMB-H solid tumors in patients who have received treatment and shown no improvement, with no other satisfactory treatment choices remaining [18]. This approval was based on a retrospective analysis conducted on 102 participants who were enrolled in the KEYNOTE-158 trial and had tumors classified as TMB-H [82]. A CR was observed in just 4% of cases, with an ORR of 29%. The median duration of response was not reached. However, half of the patients displayed response durations lasting 24 months or more. These findings suggest pembrolizumab can be a viable secondary or subsequent treatment choice for gastroesophageal tumors that are present with MSI-H/dMMR or TMB-H features. It is important to note that no patients with gastroesophageal cancer were included in the KEYNOTE-158 trial [83]. KEYNOTE-585 and KEYNOTE-811 are ongoing trials being conducted to investigate the effects of pembrolizumab on GC and GEJ [84,85]. A new contender has recently emerged as a potential first-line combination treatment for patients diagnosed with HER2-positive GC/GEJ. In the KEYNOTE 811 study, the incorporation of pembrolizumab alongside trastuzumab and CTH as first-line therapy resulted in an impressive ORR of 74% in individuals diagnosed with HER-positive metastatic GC/GEJ. However, the difference in the median duration of response was only 1.1 months compared with trastuzumab plus CTH. The long-term benefits, including PFS and OS, need further observation [86]. The results of the KEYNOTE-859 trial demonstrated that pembrolizumab in combination with CTH (capecitabine plus oxaliplatin or fluorouracil plus cisplatin) is an effective and well-tolerated first-line treatment for patients with locally advanced or metastatic HER2-negative GC or GEJ. The inclusion of pembrolizumab alongside CTH resulted in a considerable increase in OS, PFS, and ORR when contrasted with the placebo plus CTH group. The median OS in the intention-to-treat population, as well as the populations with a PD-L1 CPS of 1 or higher, and PD-L1 CPS of 10 or higher, were 2.9 months vs. 11.5 months, 13.0 months vs. 11.4 months, and 15.7 months vs. 11.8 months, respectively [87].

In April 2021, the FDA approved the use of **nivolumab**, a monoclonal PD-1 antibody, in combination with fluoropyrimidine- and platinum-based chemotherapy as a first-line treatment for advanced and metastatic GC [88]. The approval was granted on the basis of the Phase III Checkmate-649 trial that randomly assigned 1581 untreated patients, with HER2-negative and unresectable gastric, EGJ, or esophageal adenocarcinoma, to receive either chemotherapy or nivolumab with chemotherapy (capecitabine and oxaliplatin or modified FOLFOX) [88]. The study results indicated that the combination of nivolumab and chemotherapy yielded significantly higher OS (14.4 vs. 11.1 months; HR = 0.71; *p* < 0.0001) and PFS (7.7 vs. 6 months; HR = 0.68; *p* < 0.0001) rates than chemotherapy alone in patients with a PD-L1 CPS of ≥5. Additionally, it was revealed that patients with a PD-L1 CPS of ≥1 showed an improvement in OS and PFS (OS = 14 vs. 11.3 months, HR = 0.77; PFS = 7.5 vs. 6.9, HR = 0.74), and the same was observed in all randomly selected participants (OS = 13.8 vs. 11.6, HR =0.8; PFS = 7.7 vs. 6.9, HR = 0.77). A combination of nivolumab and chemotherapy resulted in 59% of patients experiencing Grade 3–4 treatment-related adverse events (AEs), compared to 44% of patients who only received chemotherapy. There were 16 treatment-related deaths in the group of patients who received nivolumab plus chemotherapy, compared to 4 in the chemotherapy-alone group. Consequently, for those with HER2-negative GC who exhibit PD-L1 expression with CPS ≥ 5, the administration of nivolumab in combination with chemotherapy based on fluoropyrimidine and oxaliplatin is proposed as the first-line treatment option [88].

**Dostarlimab-gxly**, a PD-1 inhibitor antibody, was granted FDA approval in August 2021 for the treatment of dMMR recurrent or advanced solid tumors that have progressed despite or after prior treatment, with no satisfactory alternative treatment options, and no previous exposure to a PD-1 or PD-L1 inhibitor [82]. The authorization was obtained from data collected during a non-randomized Phase I multicohort GARNET trial. Dostarlimab-gxly was assessed for its antitumor potential and safety in a trial involving 209 patients with dMMR solid tumors who had not been given PD-1, PDL-1, or cytotoxic T-lymphocyte-associated protein 4 (CTLA4) inhibitors [17,89]. The ORR was found to be 42%, comprising 9% CR and 33% PR, with a median response time of 35 months. The data indicate that dostarlimab-gxly may be a viable option for treating patients with MSI-H/dMMR gastric tumors [90].

### 5.4. Challenges and Limitations of Immune Checkpoint Inhibitor Therapy

ICIs have become a standard-of-care treatment for patients with metastatic GC and PD-L1 status is a significant marker for forecasting the effectiveness of treatment [91,92]. A significant obstacle to PD-1 and PD-L1 testing and treatment for GC is the absence of standardization in testing techniques, resulting in disparities in the assessment of PD-L1 expression and cutoffs, as well as scoring [93,94]. Additionally, the development of treatment resistance over time presents a significant challenge in the field of PD-1 and PD-L1 treatment [95]. The process leading to acquired resistance to anti-PD-1/PD-L1 therapy may entail the eventual degradation of T-cell function due to epigenetic dysfunction or the adoption of other immunosuppressive signals, an alteration in antigen presentation that lowers T-cell recognition of the tumor, and the development of resistance to the effects of interferon generated by T cells [96]. Treatment with dostarlimab-gxly was associated with AEs involving the immune system. These included pneumonitis, colitis, hepatitis, endocrine disorders, nephritis, and skin reactions [90].

## 6. Antiangiogenic Treatment for Advanced Gastric Cancer

The growth of blood vessels is a distinct characteristic of cancer, and a well-established method to hinder tumor growth involves selectively targeting and obstructing this process [97]. Clinical trials conducted over the past decade have shown that the inhibition of angiogenesis, with a particular emphasis on targeting the VEGF (vascular endothelial growth factor receptor) pathway, can boost survival rates. This is accomplished by employing mAbs that bind to VEGFA or multitarget tyrosine kinase receptor (TKR) inhibitors that have antiangiogenic specificity. Studies have shown that anti-VEGF therapies provide a survival advantage in the treatment of various types of cancers, including GC [98,99,100,101]. For this reason, approaches that result in a higher degree of VEGF signaling blockade and angiogenesis inhibition have attracted considerable attention.

### 6.1. Mechanisms of Action of Anti-VEGFR Antibodies

The main objective of anti-VEGFR treatment in advanced GC is the inhibition of the activity of VEGFR, a receptor involved in the downstream signaling pathways of angiogenesis. Angiogenesis plays a crucial role in tumor growth and metastasis, making it an attractive target for therapy. The administration of antiangiogenic treatment leads to the inhibition of new blood vessel formation, reducing the supply of oxygen and nutrients to the tumor, and ultimately suppressing tumor growth. Following the identification of VEGF family members that promote neovascularization (VEGF A, B, C, D, and E), and the discovery of various drugs that target the VEGF pathway, the mechanism of antiangiogenic therapy was verified [102,103]. Tumor cells generate VEGF, an angiogenic factor that stimulates the growth of endothelial cells [104]. It leads to an increase in the permeability of blood vessels, a reduction in endothelial cell apoptosis, the initiation of stromal proteolysis, and the promotion of endothelial cell proliferation and migration [105]. The VEGF families can bind with VEGF receptors, namely VEGFR-1, VEGFR-2, and VEGFR-3. These TKRs are found in both lymphatic and blood vessel endothelial cells. The dimerization and transphosphorylation of intracellular tyrosine kinase domains are initiated by the interaction between VEGF-A and VEGF receptor-2. This activation of tyrosine kinase enzymes and pathways ultimately promotes cellular proliferation and endothelial cell survival. The use of particular inhibitors may result in the suppression of cellular proliferation and the survival of endothelial cells [106].

### 6.2. Current Anti-VEGFR Treatment Options

The treatment potential of **ramucirumab**, a monoclonal VEGFR-2-targeting antibody, in advanced or metastatic gastroesophageal cancers has been demonstrated in two Phase III clinical trials [107,108]. The international multicenter Phase III study, REGARD, has confirmed that ramucirumab enhanced the survival rate of patients with advanced gastric or EGJ adenocarcinoma who failed to respond to first-line chemotherapy [107]. In this study, 238 participants were given ramucirumab, while the remaining 117 received the placebo, bringing the total sample size to 355. Ramucirumab treatment led to an OS of 5.2 months, in contrast to the placebo group, which demonstrated an OS of 3.8 months (*p* = 0.047).

A randomized international Phase III RAINBOW trial was undertaken to analyze the impact of paclitaxel with or without ramucirumab on 665 patients who had been diagnosed with metastatic gastric or EGJ adenocarcinoma and had progressed on first-line chemotherapy [108]. The cohort comprising 330 patients who underwent treatment with ramucirumab in addition to paclitaxel exhibited a median OS of 9.6 months, significantly surpassing the median OS of 7.3 months for the 335 patients who were administered paclitaxel monotherapy. The combination of ramucirumab and paclitaxel resulted in a median PFS of 4.4 months and an ORR of 28%. In contrast, the paclitaxel group had a median PFS of 2.8 months and an ORR of 6% (*p* = 0.0001). The exposure–response analysis disclosed that ramucirumab had a significant impact on OS and PFS in both studies [108]. Ramucirumab has received FDA approval for use as a monotherapy or in combination with paclitaxel to treat advanced gastric or EGJ adenocarcinoma that is unresponsive or progressing after first-line chemotherapy based on platinum or fluoropyrimidine.

The combination of ramucirumab and FOLFIRI offers a potential treatment option for the secondary or subsequent treatment line. A retrospective analysis was conducted to assess the outcomes of administering FOLFIRI with ramucirumab as a second-line intervention to 29 patients with advanced GC or EGJ adenocarcinoma [109]. The research findings showed an ORR of 23% and a disease control rate of 79%. According to the study results, the median PFS and OS were 6 months and 13.4 months, respectively. The OS rate was 90% and 41% after six and twelve months, respectively. The combination treatment of FOLFIRI and ramucirumab was observed to be free of any new safety concerns, thus establishing it as a reliable substitute for the ramucirumab and paclitaxel combination. For patients with advanced GC who have undergone a second or subsequent line of therapy, the combination of ramucirumab and irinotecan is a viable treatment option [110].

The international Phase III RAINFALL trial has provided evidence that ramucirumab is not effective in reducing the risk of disease progression or mortality in patients with metastatic GEJ adenocarcinoma who have not received prior treatment. Therefore, the use of ramucirumab in the first-line treatment is not recommended [111].

### 6.3. Potential Limitations of Anti-VEGFR Treatment

The promising results of clinical trials indicate that anti-VEGFR treatment has emerged as a potential therapeutic alternative for GC. However, it is important to recognize that this treatment carries certain side effects that are specific to it. Anti-VEGFR-2 antibodies often lead to hypertension as a side effect because of the involvement of VEGF in regulating blood pressure and blood vessel tone [112]. Proteinuria and hemorrhage are commonly observed side effects, with proteinuria affecting as much as 60% of patients undergoing anti-VEGFR-2 antibody therapy [113]. Although the side effects are typically manageable, they have the potential to impact the patient’s quality of life, requiring dose adjustments or even discontinuing treatment.

The resistance to treatment is an additional potential limitation of the anti-VEGFR-2 antibody therapy. The protein HIF-1 is a fundamental element in the survival of cancer cells and their adaptation to hypoxia and has been demonstrated to play a role in resistance to antiangiogenic therapy [114]. Furthermore, the upregulation of VEGFR2 in GC tissues has been recognized as a prognostic marker for poor DFS and OS. The findings imply that certain patients may exhibit resistance to anti-VEGFR-2 antibody treatment, necessitating alternative treatment approaches.

## 7. NTRK Rearrangements in Gastric Cancer

The NTRK gene, also called neurotrophic tropomyosin receptor kinase, encodes proteins that have a crucial role in the growth and development of nerve cells [115]. Nevertheless, alterations to this gene can also be a contributing factor to the onset of cancer [116]. The NTRK gene family comprises three members: NTRK1, NTRK2, and NTRK3. Each of these encodes one of three tropomyosin receptor kinases, namely TrkA, TrkB, and TrkC, respectively [117]. Research findings indicate that NTRK gene fusions cause the constitutive activation of TRK receptors, which ultimately leads to the uncontrolled transformation and proliferation of cancerous cells [117]. TRK receptors interacting with their ligands initiate the activation of signal transduction pathways involved in tumorigenesis, such as the Ras/mitogen-activated protein kinase (MAPK), the phosphatidylinositol-3-kinase (PI3K)/AKT, and the mammalian target of rapamycin (mTOR) pathways [118]. Recent studies have shown that these alterations are oncogenic drivers for several tumors within this framework [119].

### 7.1. Prevalence of NTRK Gene Alterations in Gastric Cancer

The discovery of NTRK gene fusion represents an important achievement in the realm of biomarker-targeted agnostic therapies [115,120]. Based on a global estimate in 2018, the incidence of NTRK gene fusion tumors was found to be 0.52 per 100,000 persons, and the 5-year prevalence was 1.52 per 100,000 persons [121]. In rare cancers such as secretory breast carcinoma, mammary analog secretory carcinoma, and congenital infantile fibrosarcoma, detection rates can reach as high as 90% [122]. By contrast, the detection rates for common adult cancers such as non-small cell lung, colorectal, thyroid, and salivary gland cancers are much lower. Reports suggest that NTRK gene fusions are highly uncommon in gastroesophageal cancers (about 1%) [123,124]. The NTRK gene fusion incidence in GC varies based on the type and histology of cancer. According to Pu et al.’s study in 2023, the hepatoid or enteroblastic differentiation type of GC demonstrated enrichment in NTRK gene alterations, unlike dMMR-type GC [123]. Despite its low prevalence, the identification of patients with NTRK gene fusions can aid in directing treatment decisions and improving patient outcomes. Notwithstanding this, a solitary case report furnishes proof of its existence in GC and proposes its prospective association with a more aggressive phenotype [120,125,126].

### 7.2. Detection Methods of NTRK Gene Fusions

To date, there is no FDA-approved test for the detection of NTRK gene fusion. Consequently, various methods are utilized to identify direct or indirect alterations in the NTRK genes, such as NGS, FISH, and IHC [127]. According to the current ESMO recommendations, pan-TRK immunohistochemistry is considered a viable method for screening NTRK gene fusions [128]. However, a recent study established that DNA-based NTRK fusion sequencing manifested a higher detection rate than pan-TRK IHC [129]. Moreover, modern methodologies such as liquid biopsy and Nanostring technology enable the detection of NTRK gene fusions at the DNA, RNA, or protein level, thereby making it easier to identify patients who are potential candidates for targeted therapies [130].

### 7.3. NTRK Gene Fusions as a Target for Gastric Cancer Therapy

The FDA granted accelerated approval to the TRK inhibitor **larotrectinib** in 2018 as a treatment for adults and pediatric patients who have solid tumors with an NTRK gene fusion. This authorization pertains solely to cases where there is no known acquired resistance mutation, the cancer is metastatic or surgery would likely result in harm, and where no other satisfactory treatments are available, or if the cancer has progressed after treatment [19]. The FDA’s second-ever approval of a tissue-agnostic therapy for cancer patients was grounded in information garnered from three individual clinical trials held at various centers. Individuals presenting with identified NTRK gene fusion were included in one of three protocols: a Phase I trial for adults (LOXO-TRK-14001; NCT02122913), a Phase I-II trial for children (SCOUT; NCT02637687), and a Phase II trial for adolescents and adults (NAVIGATE; NCT0257643) [131]. These trials involved the recruitment of 55 patients with inoperable or metastatic solid tumors that harbored an NTRK gene fusion and had experienced disease progression after receiving systemic treatment. The patients received larotrectinib as a therapeutic intervention. The trials indicated an ORR of 75% among participants, with 22% experiencing CR. Upon the observation of the patients over a median of 9.4 months, it was found that 86% of the responders either continued their larotrectinib treatment or underwent curative surgery. Following the one-year mark of the study’s initiation, 71% of the responses were still ongoing, and 55% of the patients had not yet experienced progression. Regarding response duration, 73% of patients had a response duration of ≥6 months, 63% of patients had a response duration of ≥9 months, and 39% of patients had a response duration of ≥12 months. The median duration of response and PFS had not been attained at the time of data analysis. The clinical trials, SCOUT and NAVIGATE are still open for the admission of patients with NTRK gene fusion-positive tumors.

The FDA authorized the use of **entrectinib**, the second TRK inhibitor, in 2019, for the same purposes as larotrectinib [20]. The approval of entrectinib for the treatment of NTRK gene fusion-positive tumors was based on three multicenter single-arm Phase I and Phase II clinical trials. The study included fifty-four adult patients with solid tumors that demonstrated an NTRK gene fusion, who were either locally advanced or metastatic. These patients were then randomly assigned to one of three protocols: ALKA-372-001, STARTRK-1 (NCT02097810), or STARTRK-2 (NCT02568267) [132]. The ORR observed across the three trials was 57%, while the rate of CR stood at 7%. A response lasting at least 6 months was observed in 68% of patients, with 45% experiencing a response that persisted for at least 12 months. The mean duration of response was 10 months. Patients diagnosed with tumors that display NTRK gene fusion are currently being recruited into the STARTRK-2 clinical trial [132].

The data suggest that entrectinib and larotrectinib can lead to persistent and clinically significant outcomes in patients with tumors positive for NTRK gene fusion while maintaining acceptable safety profiles. Thus, entrectinib and larotrectinib are recommended as second-line or subsequent therapies for individuals with NTRK gene fusion-positive GC.

The adoption of larotrectinib and entrectinib as second-line treatments for unresectable or metastatic GCs that test positive for NTRK fusion is recommended [133]. The advancement of research may lead to the growing significance of NTRK gene fusion-targeted therapy as a treatment option for GC patients. While the data from the aforementioned studies show great promise, it is important to note that they are not exclusive to the GC patient population. One must bear in mind that NTRK alterations are infrequently observed in GC.

## 8. RET Kinase Alterations in Gastric Cancer

The RET (rearranged during transfection) gene encodes an RTK and is primarily associated with oncogenic abnormalities observed in medullary thyroid cancers (70%). The germline mutations of RET are of critical importance in the development of multiple endocrine neoplasia syndromes [134]. The frequency of gene RET fusion is a common occurrence in papillary thyroid cancer (10–20%) as well as a subgroup of non-small cell lung cancer (2%), while in other solid tumors such as GC, it is typically found in less than 1% of cases [135].

In the study by Kucharczyk et al. (2022), it was found that RET displays targetable mutations and gene fusions in specific cancers, including GC [136]. The activation of the RET receptor has a significant role in embryonic development by regulating the enteric nervous system and kidneys and maintaining homeostasis in adult neural and neuroendocrine tissues under physiologic conditions [137]. The cause of the constitutive activation of RET in cancer is due to point mutations in either the extracellular or the kinase domain, or chromosomal rearrangements. It is uncommon to find RET aberrations and other significant oncogenic driver mutations coexisting [138].

### 8.1. Detection Methods of RET Gene Alterations

The determination of a testing approach for RET abnormality is based on factors such as the type of changes to be screened (fusion vs. mutation), the quality and quantity of available tissue, the number of changes that may be screened based on tumor type, and cost considerations. Multiple studies have been conducted to examine the effectiveness of RET IHC as a surrogate marker for RET activation. However, due to the lack of specific RET antibodies, there is an elevated likelihood of erroneous outcomes, and IHC is currently not recommended for the detection of RET mutations. The use of FISH enables the detection of RET rearrangements. However, its clinical efficacy is limited as a result of a high rate of false-positives and poor specificity [139]. Although qPCR techniques are quick and relatively inexpensive, their ability to recognize new fusion variations is limited, rendering them suitable only for the most prevalent aberrations. NGS is recommended as the optimal testing method due to its capacity to precisely examine multiple actionable targets concurrently. Moreover, NGS has the ability to recognize mutations, as well as both known and novel fusions, without any dependence on the fusion partner. NGS-based ctDNA testing may serve as a viable alternative when a tissue sample is unavailable [140].

### 8.2. Treatment Options for RET-Altered Gastric Cancer

Despite the low prevalence of RET fusions in GC (3.3%), they have the potential to be a therapeutic target [141]. Clinical trials are underway to investigate the treatment potential of RET inhibitors in cancers caused by RET mutations [138]. According to research, RET mutations have demonstrated a significant role in regulating cell proliferation, migration, differentiation, and survival [142]. Therefore, the targeting of RET mutations may present a promising approach toward the development of innovative treatments for GC [143,144].

**Selpercatinib**, a novel and highly selective inhibitor of RET kinase, with demonstrated CNS activity, has been proven effective in the treatment of RET fusion-positive lung and thyroid cancers. However, clinical trials are currently being conducted to assess the safety and efficacy of selpercatinib in a heterogeneous patient population with advanced solid tumors carrying RET fusion, other than in the lungs or thyroid, representing a tumor-agnostic population. The effectiveness of selpercatinib in adult patients is being examined in the ongoing Phase I/II, single-group, open-label trial LIBRETTO-001. Patients who meet the eligibility criteria are those who have experienced disease progression after previous systemic therapies or have no satisfactory therapeutic options. The primary goal was to determine the ORR as established by the independent review committee. Patients with RET fusion-positive cancer, excluding non-small-cell lung cancer and thyroid cancer, were encompassed in the efficacy-evaluable tumor-agnostic population. As of the data cutoff point, these patients had a minimum of 6 months of follow-up after the initial study dose. According to the independent review committee’s evaluation of 41 efficacy-evaluable patients, the ORR was 43.9% [145]. The efficacy of selpercatinib was clinically significant in the tumor-agnostic population with RET fusion-positive, and its safety profile remained consistent with that observed in other indications. Conducting comprehensive genomic testing, which involves identifying RET fusions, is a pivotal step for identifying patients who might benefit from selpercatinib. Currently, the study is enrolling participants and has been registered with ClinicalTrials.gov under NCT03157128 [145].

The ARROW clinical trial (NCT03037385) was planned to assess the effectiveness and safety of **pralsetinib** in patients with advanced RET-altered solid tumors, including GC. The potency of pralsetinib lies in its ability to selectively inhibit RET kinases, including RET fusion proteins. The ORR served as the main endpoint in Phase II of this study. Within the cohort of 23 eligible patients for efficacy analyses, a 57% ORR was observed. A confirmed CR was identified in 13% of the patients, and a confirmed PR was observed in 43%. These findings validate RET as a tissue-agnostic target, underscoring the potential of pralsetinib as a well-tolerated treatment option for patients with solid tumors harboring RET fusion [146].

Preclinical and clinical studies have confirmed the promise of emerging therapeutic strategies targeting RET fusions in GC. RET inhibitors have exhibited the ability to address genetic abnormalities arising from RET mutations and rearrangements, offering a viable treatment alternative for GC patients [138].

The clinical significance of RET modifications has increased following the integration of two notable new drugs into precision oncology resources. Nonetheless, it is important to mention that the data from the LIBRETTO-001 and ARROW studies do not specifically pertain to the GC patient population. At present, there is increased attention to the advantages of RET-specific TKIs in the initial treatment stage. In keeping with the recommended strategy, genomic sequencing should be conducted at the time of diagnosis, and selective RET inhibitors should be started at an earlier stage.

## 9. BRAF Mutation in Gastric Cancer

The RAS/RAF/MEK/ERK pathway, or the mitogen-activated protein kinase (MAPK) pathway, holds great significance in the realm of cancer biology. A range of cellular functions are affected by this pathway, including proliferation, migration, survival, angiogenesis, and cell cycle regulation [147]. The possibility of the consistent activation of the pathway is attributed to mutations in the BRAF proto-oncogene, a serine/threonine kinase, which is found in various types of cancer [148]. The proto-oncogene BRAF facilitates ERK signaling, advances cell growth, and promotes cellular transformation [149]. The most commonly occurring BRAF alteration, which is predominantly prevalent in melanoma, is the V600E mutation, characterized by substituting valine (V) with glutamic acid (E) at amino acid sequence position 600 [150]. The function of BRAF is altered by the V600E mutation, which disrupts hydrophobic interaction, leading to the folding of the BRAF kinase into an active catalytic configuration [151]. Additionally, this mutation is associated with highly aggressive behavior in multiple cancer types [152,153]. Although BRAF mutations are rare in GC, several studies have documented incidence rates of BRAF V600E mutation of up to 12% among GC patients [154].

### BRAF V600E Mutation as a Target in Gastric Cancer Treatment

The FDA has approved the use of **dabrafenib** in combination with **trametinib** for the treatment of solid tumors that are unresectable or metastatic and positive for the BRAFV600E mutation. This combination has been authorized for patients aged 6 years and above, who had tumor progression after prior therapy and have no alternative treatment options. The approval’s validation was based on the significant efficacy and safety of the combination, as demonstrated by the Rare Oncology Agnostic Research (ROAR) [155] and National Cancer Institute—Molecular Analysis for Therapy Choice [156] (NCI-MATCH, NCT02465060) studies conducted on adults, as well as a study (NCT02124772) conducted on pediatric patients with refractory or recurrent solid tumors. The recent authorization for a tumor-agnostic approach that employs a BRAF and MEK inhibitor combination marks a significant leap forward in precision medicine. This approach leads to a more sustained response, a delay in the emergence of resistance, and a reduction in the frequency of hyperproliferative lesions in contrast to BRAF monotherapy inhibition [157].

Dabrafenib has a specific inhibitory effect on the mutated BRAF kinase, while trametinib selectively and reversibly inhibits the activation and kinase activity of MEK1 and MEK2 through an allosteric mechanism. The combination therapy of dabrafenib and trametinib hinders oncogenic MAPK pathway signaling, inhibits the proliferation and viability of BRAFV600-mutant cells, and amplifies the antitumor potential beyond monotherapy [158]. The use of this combined therapy has been approved for the treatment of BRAFV600E anchor tumor categories [159,160,161]. Despite the frequent occurrence of BRAFV600E mutations in over 40 tumor types, there is an ongoing deficiency in treatment options for rare cancers that exhibit this mutation in adults and children alike [162].

The ROAR study is a prospective study focused on investigating a combined therapeutic strategy involving BRAF and MEK inhibitors for patients who have advanced rare cancers with the BRAFV600E mutation [163]. The ROAR study has provided evidence for the pan-cancer efficacy of the BRAF and MEK inhibitor combination in 21 histologies. The ROAR study involved patients diagnosed with advanced disease, all of whom had previously undergone standard-of-care therapies [163].

Following the initiation of the ROAR study, the NCI commenced the MATCH study to evaluate dabrafenib plus trametinib in diverse BRAFV600E-mutated solid and hematological malignancies [156]. In patients with BRAF V600E/D/R/K mutation-positive solid tumors, lymphomas, or multiple myeloma whose disease had progressed on at least one standard therapy, the NCI-MATCH study reported a 38% ORR (90% CI: 22.9, 54.9; *p*  <  0.0001) with dabrafenib plus trametinib [156].

The combination treatment with dabrafenib plus trametinib showed meaningful clinical activity in patients with BRAFV600E-mutated rare cancers and was approved as a therapeutic option in patients with advanced, rare solid tumors with BRAFV600E mutations. The clinical practice of testing for BRAFV600E mutations can enhance outcomes by offering a targeted treatment option for patients with rare cancers and restricted treatment alternatives. The prompt identification of eligible patients for BRAFV600E-targeted treatment is crucial, requiring the timely implementation of genetic testing and tumor profiling in the management plan.

The selection of of approved therapeutic agents are presented in Table 1 and Table 2.

## 10. Future Considerations

Several ongoing trials evaluating new molecules or multidrug regimens in advanced GC warrant particular attention (Table 3). Two current studies aim to address inquiries concerning issues and conclusions arising from the registration studies of **ramucirumab**. The RAMIRIS study was conducted to address a significant inquiry that arose from the 2019 retrospective study by Klempner et al. [109]. It aims to evaluate the efficacy of ramucirumab with the FOLFIRI regimen in contrast to the commonly used combination of ramucirumab and paclitaxel as a second-line treatment for patients with advanced or metastatic GC. The objective of the ARMANI study was to determine whether early switching to ramucirumab and paclitaxel is a viable option, given that the RAINFALL study had ruled out the possibility of using ramucirumab in first-line treatment [111]. This research aims to determine whether the timely delivery of an active, non-cross-resistant second-line regimen (ramucirumab plus paclitaxel) can lead to improved PFS and quality of life. The suggested method may be beneficial for patients who are ineligible for second-line therapy due to the rapid deterioration of their health status following the initial disease progression.

An important concern in the cohort of patients with HER2-positive GC is the inadequacy of treatment alternatives after the initial trastuzumab therapy, whether due to resistance or progression during the treatment. In the metastatic stage, the majority of patients suffer tumor progression approximately 8 months after receiving the first-line trastuzumab-based treatment [24]. If receptor conversion is absent, patients require the continuation of anti-HER2 therapy to manage the disease. The ongoing trials aim to evaluate newly discovered molecules as potential treatment options. **KN026**, a bispecific anti-HER2 antibody, exhibits a noteworthy capacity to surpass trastuzumab resistance. Its design allowed for the simultaneous bindings of HER2 domains II (pertuzumab binding site) and IV (trastuzumab binding site) and inherited trastuzumab’s antibody-dependent cellular cytotoxicity (ADCC) and phagocytic killing effect. KN026 demonstrated a favorable safety profile and improved ORR as a second-line therapy for advanced HER2-positive GC/GEJ patients [164].

Xu et al. [165] assessed KN026 in a multicenter Phase II study on the Chinese population and established two cohorts based on high- and low-level HER2 expression. Patients diagnosed with HER2-positive GC demonstrated an ORR of 56% (95% CI 35–76%), with a response duration of 9.7 months (95% CI 4.2—not evaluable). The treatment used did not result in an increase in typical cardiotoxic complications [165]. Clinical trials, such as GATSBY [166], investigating trastuzumab emtansine, and T-ACT [46], investigating trastuzumab with paclitaxel, have emphasized the importance of continuing anti-HER2 therapy. By failing to achieve primary efficacy endpoints, these trials have underscored the challenge of resistance to first-line anti-HER2 therapy. The complete blockade of the HER2 signaling pathway through dual-antibody therapy or bispecific antibody has the potential to mitigate primary and overcome acquired resistance following trastuzumab treatment. In principle, the biparatopic antibody KN026 may possess a few advantages over pertuzumab and trastuzumab combination [167]. Although possessing the same molecular target, the application of dual-HER blockade through trastuzumab and pertuzumab (JACOB trial) [168] did not achieve the same remarkable results found in breast cancer. KN026 has the potential to provide a more robust receptor-clustering effect than monospecific antibodies, which may promote rapid receptor internalization and signal downregulation. KN026 showed promising efficacy outcomes in the further-line setting by targeting potential resistance mechanisms and improving the drug potency. Advanced HER2-positive GC/GEJ patients who progressed on prior trastuzumab treatment showed promising results with an ORR of 50%, a median PFS of 5.5 months, and a median OS of 14.9 months [165].

KN026, a cytotoxic drug-free option, was associated with better safety outcomes, with only 20% of patients experiencing SAEs compared to the current second-line treatment option—trastuzumab deruxtecan [169,170]. A Phase II–III study of KN026 combined with CTH versus CTH alone in the second-line treatment of HER2-positive advanced or metastatic GC has been launched recently (NCT05427383) to further investigate the potential of KN026 in combination therapy [165,169].

It is worth noting that research evaluating the utilization of the KN026 and KN046 combination has granted this combination the orphan drug designation [169,171].

**Disitamab vedotin** (**RC48**), a HER2-targeted antibody–drug conjugate containing monomethyl auristatin E (MMAE), is one of the novel molecules currently undergoing clinical evaluation for HER2-positive GC patients. However, the ORR (25%) and duration of response (5 months) do not seem to exceed that of trastuzumab deruxtecan, and SAEs were less common (36% vs. 44%) [33,47,172]. Conditional marketing approval has been granted to RC48 in China as a third-line treatment for advanced HER2-positive GC, and it could potentially serve as a feasible option for subsequent treatment lines [33,172].

**Zanidatamab** (**ZW25**) is a humanized, bispecific monoclonal anti-HER2 antibody (targeting 2 distinct HER2 epitopes—ECDII and ECDIV). Zanidatamab can impede the growth of human cancer cell lines, irrespective of their HER2 expression levels. Furthermore, it has been observed that the drug exhibits a sustainable, cumulative, and synergistic effect when paired with multiple chemotherapeutic agents, including platins, taxanes, microtubule inhibitors, and DNA synthesis inhibitors [173]. In addition, zanidatamab was assessed as an initial treatment option for individuals with advanced gastroesophageal cancer, administered concurrently with CTH. The preliminary findings are highly encouraging, showing a survival rate of 87% at 18 months [174].

Among the known molecular targeted therapies, the family of ICI drugs is also growing. **Tislelizumab** is a humanized IgG4 anti-PD-1 monoclonal antibody specifically designed to minimize binding to FcγR on macrophages. This drug was designated an orphan drug. The RATIONALE 305 study demonstrated the efficacy of tislelizumab in combination with CTH as a first-line treatment for GC/GEJ, resulting in an OS of 17.2 months compared to 12.6 months in the placebo plus CTH group [167,175].

**Zimberelimab**, a monoclonal immunoglobulin G4 that specifically targets the PD-1 receptor, has been recently developed and is currently undergoing safety and tolerability studies for multiple types of cancer [176,177]. The coinhibition of PD-1/PD-L1 and TGF-β pathways is a promising therapeutic approach to cancer treatment. **SHR-1701**, a new bifunctional fusion protein, is a combination of a monoclonal anti-PD-L1 antibody and the extracellular domain of TGF-β receptor II. A satisfactory safety profile and promising efficacy were observed in previously treated advanced solid tumors, particularly in GC, upon administering SHR-1701, with an ORR of 20% (95% CI, 8.4–36.9) and a 12-month OS of 54% (95% CI, 29.5–73.9) [178].

The final analysis of the Phase III ORIENT-16 trial, which aimed to evaluate the effectiveness of **sintilimab** (a PD-1 inhibitor) compared to the placebo plus CTH as a first-line treatment for patients with advanced GC and GEJ, demonstrated a significant OS benefit in patients with CPS ≥5 who received sintilimab (19.2 months versus 12.9 months), as compared to the control group [179].

**Avelumab** is another anti-PD-L1 antibody that is being under investigation [180]. A randomized Phase III clinical trial called JAVELIN Gastric 300 (NCT02625623) compared the use of third-line avelumab versus CTH. However, this study did not meet its primary endpoint of improving OS (4.6 vs. 5.0 months) in avelumab vs. CTH arms. The secondary endpoints were met for neither PFS nor ORR [181]. The JAVELIN Gastric 100 (NCT02625610) clinical trial evaluated the effectiveness of switch-maintenance treatment. This clinical trial aims to test if avelumab can provide lasting antitumor activity after tumor shrinkage and immunogenic priming resulting from first-line CTH, with reduced tumor toxicity due to additional CTH. The study results indicated that avelumab maintenance did not demonstrate superior OS outcomes compared to continued CTH in patients with advanced GC or GEJ, both in the overall population and in a predefined PD-L1-positive subgroup [182].

Despite the numerous molecular targets identified in the pathogenesis of GC, new ones continue to emerge to address the persistent need for advancements in treatment outcomes. Various new targeted therapies are currently under evaluation.

The anti-TIGIT (T-cell immunoglobulin and immunoreceptor tyrosine-based inhibitory motif (ITIM) domain) monoclonal antibody, **domvanalimab**, which is currently the most advanced in clinical trials, has displayed encouraging outcomes as a strategy when used in conjunction with anti-PD-1/PD-L1 therapies. TIGIT binding is the mechanism through which domvanalimab functions, with the potential to activate the immune system for the purpose of eliminating cancer cells [183].

**Bemarituzumab** is a humanized monoclonal antibody of the IgG1 isotype, which has been specifically engineered to target the fibroblast growth factor receptor 2b (FGFR2b) in tumors featuring FGFR2 gene amplification and FGFR2b overexpression [184,185]. Approximately 30% of non-HER2-positive GC [186,187] display FGFR2b overexpression. Bemarituzumab operates through two fundamental mechanisms of action. The involved mechanisms encompass binding to FGFR2b, which leads to the inhibition of FGF signaling, along with receptor downregulation, internalization, and degradation, and an escalation in ADCC. The FIGHT-FPA144 study shows that the application of bemarituzumab in immunochemotherapy results in a substantial improvement in PFS (9.5 vs. 7.4 months HR, 0.68) and OS (19.2 vs. 13.5 months HR, 0.60; 95% CI, 0.38–0.94) for patients with at least 10% FGFR2b overexpression in tumor cells. Bemarituzumab was granted the Breakthrough Therapy Designation based on the findings of the FIGHT–FPA144 study [184]. Presently, the study FORTITUDE-101 (NCT05052801), evaluating mFOLFOX-6 and nivolumab with vs. without bemarituzumab, is underway [188].

**Evorpacept**, a myeloid ICI, has shown an impressive capability to inhibit integrin-associated protein CD47 effectively with high affinity. Moreover, the inactive Fc region can safely enhance the anticancer therapeutics. It has been confirmed that evorpacept is an effective and safe second-line treatment option for advanced HER2-positive GC patients. An ORR of 38.5% was observed along with a mPFS of 5.6 months and an estimated one-year OS rate of 83.3% (ORR 72.2%, mPFS of 9.8 months; estimated 12-month OS, 77.7%) [178]. According to the interim efficacy results of the ASPEN-06 trial, the combination treatment of evorpacept demonstrated a confirmed ORR of 52%, in contrast to the control group receiving trastuzumab + CYRAMZA + paclitaxel, which achieved an ORR of 22% [189].

**Zolbetuximab** is an innovative immunoglobulin G1 monoclonal antibody that specifically targets claudin-18 isoform 2 (CLDN18.2) and facilitates antibody-dependent cellular cytotoxicity and complement-dependent cytotoxicity in CLDN18.2-positive GC and GEJ cells. The Phase III study GLOW (NCT03653507) investigated the efficacy of zolbetuximab in combination with CAPOX as a first-line treatment for patients with CLDN18.2-positive, HER2-negative, locally advanced unresectable or metastatic GC and GEJ. GLOW successfully achieved the primary endpoint of PFS (8.21 months with zolbetuximab versus placebo’s 6.80 months), as well as the key secondary endpoint of OS (14.39 months versus 12.16 months). The combination of zolbetuximab and CAPOX has the potential to serve as a novel first-line treatment option for patients diagnosed with CLDN18.2-positive, HER2-negative, locally advanced unresectable or metastatic GC, and GEJ [190].

**Table 3:** Selection of ongoing trials evaluating new molecules or multidrug regimens in advanced GC.

Study Name	KN026 in Combination with Chemotherapy in the Second Line Treatment of HER-2-Positive Advanced or Metastatic Gastric Cancer	A Study of RC48-ADC in Local Advanced or Metastatic Gastric Cancer with the HER2-Overexpression	A Study of Zanidatamab in Combination with Chemotherapy Plus or Minus Tislelizumab in Patients with HER2-Positive Advanced or Metastatic Gastric and Esophageal Cancers (HERIZON-GEA-01)	Study of Tislelizumab in Combination with SOX for the Treatment of Gastric Cancer with Liver Metastases	Safety and Efficacy of Sintilimab in Combination with Chemoradiotherapy Followed by D2 Surgical Resection in Patients with Advanced Gastric Cancer with Retroperitoneal Lymph Node Metastasis	HX008 Plus Irinotecan Versus Placebo Plus Irinotecan as Second-Line Treatment in Advanced Gastric Cancer	A Study to Evaluate the Efficacy and Safety of ONO-4538 in Combination with Ipilimumab and Chemotherapy in Chemotherapy-naïve Participants with HER2-Negative Unresectable Advanced or Recurrent Gastric Cancer
ClinicalTrials.gov Identifier:	NCT05427383	NCT04714190	NCT05152147	NCT05325528	NCT05002686	NCT04486651	NCT05144854
Therapeutic agent	KN026 [165]	RC48-ADC [172]	Zanidatamab [174]	Tislelizumab [167]	Sintilimab [179]	HX008 [191]	ONO-4538 [192]
Classification	anti-HER2 bispecific antibody	anti-HER2 monoclonal antibody-Monomethyl auristatin E (MMAE) conjugate	anti-HER2 antibody and PD-1 monoclonal antibody	PD-1 monoclonal antibody	PD-1 monoclonal antibody	PD-1 monoclonal antibody	PD-1 monoclonal antibody and anti-CTLA-4 monoclonal antibody
Phase	Phase II, Phase III	Phase III	Phase III	Phase II, Phase III	Phase II, Phase III	Phase III	Phase III
Population	patients with HER-2-positive advanced or metastatic GC	patients with HER-2-positive locally advanced or metastatic GC	patients with HER-2-positive advanced or metastatic GC and EC	patients with GC with liver metastases	patients with advanced GC with retroperitoneal lymph node metastasis	patients with advanced GC or GEJ adenocarcinoma who have had tumor progression after first-line treatment with platinum and/or fluoropyrimidine therapy	chemotherapy-naïve participants with HER2-negative unresectable advanced or recurrent GC
Study arm	KN026 + chemotherapy	RC48-ADC	zanidatamab plus CTH with or without tislelizumab	Tislelizumab in combination with SOX (Tegafur + Oxaliplatin)	Sintilimab + chemoradiotherapy followed by D2 surgical resection	HX008 + irinotecan	ONO-4538 + ipilimumab + CTH
Control arm	placebo + CTH	physician choice standard treatment	standard of care (trastuzumab plus CTH)	NA	NA	placebo + irinotecan	CTH
Recruitment status	Recruiting	Recruiting	Recruiting	Recruiting	Recruiting	Recruiting	Recruiting
Primary outcome measures:	PFS according to RECIST 1.1 OS according to RECIST 1.1	OS	PFS by BICR OS	ORR	1 year PFS	OS in All Participants OS)in Participants with PD-L1 CPS ≥ 1	OS
Study Name	A Trial of SHR1701 Plus Chemotherapy in Patients with Gastric or Gastroesophageal Cancer	A Clinical Trial of a New Combination Treatment, Domvanalimab and Zimberelimab, Plus Chemotherapy, for People with an Upper Gastrointestinal Tract Cancer That Cannot be Removed with Surgery That Has Spread to Other Parts of the Body (STAR-221)	RegoNivo vs. Standard of Care Chemotherapy in AGOC (INTEGRATEIIb)	Ramucirumab Plus FOLFIRI Versus Ramucirumab Plus Paclitaxel in Patients with Advanced or Metastatic Gastric Cancer, Who Failed One Prior Line of Palliative Chemotherapy (RAMIRIS)	Assessment of Ramucirumab Plus Paclitaxel as the Maintenance Versus Continuation of First-line Chemotherapy in Patients with Advanced HER-2 Negative Gastric or Gastroesophageal Junction Cancers (ARMANI)	Bemarituzumab Plus Chemotherapy and Nivolumab Versus Chemotherapy and Nivolumab for FGFR2b Overexpressed Untreated Advanced Gastric and Gastroesophageal Junction Cancer (FORTITUDE-102)	A Study of Evorpacept (ALX148) in Patients with Advanced HER2+ Gastric Cancer (ASPEN-06)
ClinicalTrials.gov Identifier:	NCT04950322	NCT05568095	NCT04879368	NCT03081143	NCT02934464	NCT05111626	NCT05002127
Therapeutic agent	SHR-1701	Domvanalimab, Zimberelimab	Regorafenib and Nivolumab	Ramucirumab	Ramucirumab	Bemarituzumab	Evorpacept
Classification	Bifunctional anti-PD-L1/TGF-βRII agent	anti-TIGIT monoclonal antibody and PD-1 monoclonal antibody	multitargeted tyrosine kinase inhibitor and PD-1 monoclonal antibody	anti-VEGFR monoclonal antibody	anti-VEGFR monoclonal antibody	Anti-FGFR2b monoclonal antibody	CD47 antigen inhibitor
Phase	Phase III	Phase III	Phase III	Phase II, Phase III	Phase III	Phase III	Phase II, Phase III
Population	Patients with previously untreated, advanced, or metastatic GC or GEJ cancer	participants with locally advanced unresectable or metastatic GC, EC, GEJ cancer	Patients with refractory advanced GEJ cancer	Patients with advanced or metastatic GC, who failed one prior line of palliative CTH	Patients with unresectable locally advanced or metastatic HER-2 negative GC or GEJ cancer, without disease progression, following 3 months of first-line doublet CTH	Patients with FGFR2b overexpressed untreated advanced GC and GEJ Cancer	Patients with metastatic HER2-overexpressing GC and GEJ cancer that has progressed on or after prior HER2-directed therapy and fluoropyrimidine- or platinum-containing chemotherapy and are suitable for chemotherapy (2nd-line or 3rd-line)
Study arm	SHR-1701 + CAPOX	Domvanalimab + zimberelimab + multiagent CTH	regorafenib + nivolumab	ramucirumab + FOLFIRI	ramucirumab plus paclitaxel, given as switch maintenance,	bemarituzumab + mFOLFOX6 + nivolumab	Evorpacept + trastuzumab + ramucirumab + paclitaxel
Control arm	Placebo + CAPOX	nivolumab + multiagent CTH	current standard CTH options	paclitaxel + ramucirumab	continuation of first-line chemotherapy, given as per standard clinical practice	placebo plus mFOLFOX6 and nivolumab	Ramucirumab + paclitaxel
Recruitment status	Recruiting	Recruiting	Recruiting	Recruiting	Recruiting	Recruiting	Recruiting
Primary outcome measures:	AEs and SAEs in part 1 study PFS in part 2 study assessed based on BICR OS in part 2 study	OS	OS	OS rate at 6 months for Phase II OS and ORR for Phase III	PFS	OS in FGFR2b ≥ 10% 2+/3+ Tumor Cell Staining Participants	ORR per RECIST 1.1 OS

CTH—chemotherapy, RECIST—response evaluation criteria in solid tumor, GC—gastric cancer, EC—esophageal cancer, PFS—progression-free survival, OS—overall survival, ORR—objective response rate, CPS—combined positive score, GEJ—gastroesophageal junction cancer, CTLA-4—cytotoxic T-lymphocyte-associated antigen 4, BICR—blinded independent central review, NA—not applicable.

## 11. Conclusions

Over the past few years, there has been a significant acceleration in the development of therapies for advanced GC. The identification of new molecules and molecular targets is expanding our understanding of the disease’s intricate nature. Additional studies incorporating regimens with established clinical efficacy will be instrumental in managing these novel aspects of GC. The end of the classical oncology era, which relied on well-studied chemotherapeutic agents, is giving rise to novel and unexplored challenges, which will cause a significant transformation of the current oncological knowledge in the next few years. The hitherto consistent knowledge of a recognized disease is fragmenting. The verification of the presence of specific molecular targets can condition therapeutic decisions that may significantly impact treatment outcomes. The treatment of advanced GC is poised to enter a new era. For the optimal management of new therapies, the development of regimens is required, and alternative therapies must be developed for continued treatment if first-line therapy fails. The overexpression of HER2 in GC, although the most extensively researched therapeutic target, still poses various unknowns, with alternative options to trastuzumab therapy under development. Trastuzumab’s example emphasizes that the results of studies on other diseases cannot be extrapolated.

Undoubtedly, targeted therapy represents the future of oncology treatment. Although numerous therapies have gained recognition within the oncological community, there is still ample opportunity for enhancement. A definite disadvantage of targeted therapy lies in its emphasis on particular tumor traits, resulting in a substantial number of advanced gastrointestinal cancer patients being excluded from the ambit of most new medicinal drugs. Ramucirumab and other antiangiogenic drugs are distinguished from other targeted therapies due to their molecular target being ubiquitous in the patient population. As the molecular targets of the remaining therapies are present in only a small number of patients, the resulting tumor subtypes frequently meet the criteria for rare diseases.

It is imperative to consider that resistance to therapy may develop during treatment or at the outset, adding to the complexity of the issue. The application and effectiveness of any treatment can be considerably influenced by this. Therefore, given this advancement, it is imperative to establish a comprehensive collection of targeted therapies that can be implemented in the succeeding treatment phases to conquer the emerging treatment resistance. This approach represents the only viable option for affording advanced GC patients access to subsequent lines of treatment in the face of PD, thereby providing hope for an improved survival outcome.

## Figures and Tables

**Table 1:** Selection of approved therapeutic agents in advanced GC treatment.

Therapeutic Agent	Trastuzumab		Fam-Trastuzumab Deruxtecan-Nxki	Ramucirumab		Nivolumab
Study name	ToGA trial [24]	HERXO [44]	DESTINY-Gastric01 [47]	REGARD [107]	RAINBOW [108]	Checkmate-649 [88]
Phase	Phase III	Phase II	Phase II	Phase III	Phase III	Phase III
Population	patients with HER2-overexpression-positive advanced gastric or GEJ adenocarcinoma	the first-line treatment of patients with HER2 overexpression-positive advanced gastric or GEJ adenocarcinoma	advanced or metastatic gastric or GEJ adenocarcinoma in patients with progressive disease following at least two prior lines of therapy, including trastuzumab	patients with advanced gastric or GEJ adenocarcinoma progressing after first-line chemotherapy	patients with metastatic gastric or GEJ adenocarcinoma progressing on first-line chemotherapy	patients with previously untreated, HER2-negative, unresectable gastric, GEJ, or esophageal adenocarcinoma
Study arm	trastuzumab + CTH	trastuzumab + CTH	fam-trastuzumab deruxtecan-nxki	ramucirumab	ramucirumab + paclitaxel	nivolumab + CTH
Control arm	CTH alone	Single arm	CTH alone	placebo	paclitaxel alone	CTH alone
Randomization	1:1	no	2:1	1:1	1:1	1:1
Number of patients	594	45	188	355	665	1581
Follow-up time (months)	19 (study group), 17 (control group)	13.7				
OS (months)	All patients: 13.8 vs. 11 A post hoc subgroup analysis: IHC 2+ and FISH positive or IHC 3+: 16 vs. 11.8 IHC 0 or 1+ and FISH positive: 10 vs. 8.7	13.8	12.5 vs. 8.4	5.2 vs. 3.8	9.63 vs. 7.36	All patients: 13.8 vs. 11.6 PD-L1 CPS of ≥5: 14.4 vs. 11.1 PD-L1 CPS of ≥1: 14 vs. 11.3
PFS (months)		7.1	5.6 vs. 3.5		4.4 vs. 2.86	All patients: 7.7 vs. 6.9 PD-L1 CPS of ≥5: 7.7 vs. 6 PD-L1 CPS of ≥1: 7.5 vs. 6.9
ORR			40.5% vs. 11%		28% vs. 6%	

CTH—chemotherapy, PFS—progression-free survival, OS—overall survival, ORR—objective response rate, CPS—combined positive score, GEJ—gastroesophageal junction cancer, HER—human epidermal growth factor receptor, IHC—immunohistochemistry, FISH—fluorescence in situ hybridization, PDL-1—programmed death-1.

**Table 2:** Selection of tissue-agnostic approved therapeutic agents in advanced GC treatment.

		Pembrolizumab		Dostarlimab-Gxly	Larotrectinib	Entrectinib
Classification		PD-1 antibody		anti-PD-1 antibody	TRK inhibitor	TRK inhibitor
FDA approval date		2017 (first-ever tissue- and site-agnostic approval)	2020	2021	2018 (second-ever tissue-agnostic approval)	2019
Indication		treatment of patients with unresectable or metastatic MSI-H or dMMR solid tumors that have progressed following prior treatment and who have no satisfactory alternative treatment options	treatment of patients with metastatic TMB-H solid tumors who have progressed following prior treatment and who have no satisfactory alternative treatment options	treatment of patients with dMMR recurrent or advanced solid tumors that have progressed on or following prior treatment, who have no satisfactory alternative treatment options, and who had not previously received a PD-1 or PD-L1 inhibitor.	treatment of adult and pediatric patients (aged 12 years and older) with solid tumors that have an NTRK gene fusion without a known acquired resistance mutation, that are either metastatic or where surgical resection is likely to result in severe morbidity, and who have no satisfactory alternative treatments or whose cancer has progressed following treatment	the same indications as larotrectinib, as well as for adult patients with metastatic NSCLC whose tumors are ROS1-positive
Clinical trials	Protocol name		KEYNOTE-158 [81]	GARNET [89]	LOXO-TRK-14001 [131] SCOUT NAVIGATE	ALKA-372-001 [132] STARTRK-1 STARTRK-2
	Number of patients	149	102	209	55	54
	Basis of granting approval by FDA	based on data from patients with MSI-H/dMMR cancers enrolled across five multicenter single-arm clinical trials	based on a retrospective analysis of patients who had tumors identified as TMB-H	based on the nonrandomized Phase I multicohort trial that evaluated the safety and antitumor activity of dostarlimab-gxly in patients with dMMR solid tumors who had not received prior PD-1, PDL-1, or CTLA4 inhibitors.	based on data from three multicenter single-arm clinical trials enrolling patients with unresectable or metastatic solid tumors harboring an *NTRK* gene fusion who experienced disease progression following systemic therapy	based on data from three multicenter single-arm Phase I and Phase II clinical trials enrolling patients aged 18 years or older with metastatic or locally advanced *NTRK* gene fusion-positive solid tumors
	Cancer type	90 patients had colorectal cancer		The majority of patients had endometrial or GI cancers.	The most common cancer types represented were salivary gland tumors (22%), soft tissue sarcoma (20%), infantile fibrosarcoma (13%), and thyroid cancer (9%)	The most common cancer types represented were sarcoma, NSCLC, mammary analog secretory carcinoma, breast, thyroid, and colorectal
ORR		39.6%	29%	42%	75%	57%
Type of response		11 complete responses and 48 partial responses	4% complete response rate	9% complete response rate and 33% partial response rate	complete response rate of 22%.	complete response rate of 7%.
Duration of response		responses lasted ≥6 months for 78% of those who responded to pembrolizumab	the median duration of response was not reached, with 50% of patients having response durations of ≥24 months	the median duration of response was 35 months.	At 1 year, 71% of the responses were ongoing and 55% of the patients remained progression-free. Response duration was ≥6 months for 73%, ≥9 months for 63%, and ≥12 months for 39% of patients. At the time of data analysis, the median duration of response and PFS had not been reached.	Response duration was ≥6 months for 68% of patients and ≥12 months for 45% of patients. The median duration of response was 10 months.

CTH—chemotherapy, PFS—progression-free survival, OS—overall survival, ORR—objective response rate, GI—gastrointestinal, CTLA-4—cytotoxic T-lymphocyte-associated antigen 4, PDL-1—programmed death-1, dMMR—deficient mismatch repair, MSI-H—microsatellite instability (high), NSCLC—non-small cell lung cancer, ROS1—ROS proto-oncogene 1, NTRK—neurotrophic tropomyosin receptor kinase.

## Data Availability

No new data were created or analyzed in this study. Data sharing is not applicable to this article.
